# Non-Invasive Cardiac and Vascular Monitoring in Systemic Sclerosis: Impact of Therapy on Subclinical Dysfunction

**DOI:** 10.3390/medicina60122080

**Published:** 2024-12-19

**Authors:** Ștefania Lucia Magda, Ana Maria Gheorghiu, Raluca Ileana Mincu, Andrea Olivia Ciobanu, Tudor Constantinescu, Elisa Cristina Popa, Carina Mihai, Dragoș Vinereanu

**Affiliations:** 1 Department of Cardiology and Cardiovascular Surgery, “Carol Davila” University of Medicine and Pharmacy, 020021 Bucharest, Romania; 2 Department of Cardiology and Cardiovascular Surgery, University and Emergency Hospital, 050098 Bucharest, Romania; 3 Department of Rheumatology, Clinical Hospital “Dr. Ioan Cantacuzino”, 050474 Bucharest, Romania; 4 Department of Cardiology and Vascular Medicine, West German Heart and Vascular Medicine Center, 45147 Essen, Germany; 5 Institute of Pneumology “Marius Nasta”, 050159 Bucharest, Romania; 6 Department of Rheumatology, University Hospital Zürich, University of Zürich, 8091 Zürich, Switzerland

**Keywords:** treated systemic sclerosis, subclinical systolic dysfunction, echocardiography, arterial stiffness

## Abstract

*Background and objectives*: Systemic sclerosis (SSc) causes myocardial and microvascular impairment, with subclinical dysfunction and eventually permanent cardio-vascular damage. The long-term influence of SSc therapies on subclinical cardiovascular dysfunction is insufficiently investigated. We aimed to assess 2D and 4D cardiac ultrasound parameters of heart function in patients with different forms of SSc versus controls and to determine the evolution of cardiac function and arterial stiffness parameters under therapy. *Materials and methods*: A total of 60 subjects with SSc were studied at baseline; 30 SSc patients were compared to 30 matched controls. A total of 52 SSc subjects were reassessed after 1 year and 30 after 2 years of treatment. Cardiac function was evaluated through 2D standard echocardiography, tissue Doppler, speckle tracking and 4D auto LV quantification echo. Arterial stiffness was determined via the cardio-ankle vascular index and ankle brachial index. *Results*: At baseline, the standard echo parameters were normal. The 4D and myocardial work parameters, although in normal limits, were significantly altered in the SSc group vs. controls (4D ejection fraction 54.5 ± 8.5% in SSc vs. 63.8 ± 3.1% in controls; 4D longitudinal strain −14.2 ± 2.4% in SSc vs. −22.0 ± 2.7% in controls; global constructive work 2124.2 ± 449.5 mmHg% in SSc vs. 3102.8 ± 337.5 mmHg% in controls, for all *p* ≤ 0.02). Both at 1 year and 2 years of treatment, all echo and arterial stiffness parameters were similar to baseline, with no correlation to treatment type. *Conclusions*: SSc determines subclinical systolic dysfunction. Non-invasive assessment methods do not detect a functional cardiovascular decline in patients on classical therapy. Complex cardiac follow-up should be implemented in cases at risk for complications.

## 1. Introduction

Systemic sclerosis (SSc) is a systemic autoimmune disease with chronic evolution and multiple system involvement via microvasculopathy and fibrosis that causes significant morbidity and mortality. The global incidence of SSc varies from 8 to 56 per million persons annually, while the prevalence rates are between 38 and 341 cases per million persons [1]. Higher incidence and prevalence rates were noted in females (female to male ratio 5:1), adults, and developed countries [2].

Due to improvement in the prognosis of renal scleroderma crisis, cardiac and pulmonary disease are now the main mortality triggers in SSc. Cardiovascular mortality may reach 20% at 10 years from diagnosis, with maximal impact in the first 5 years (mortality rate 14%) [3].

There are two main clinical subsets of SSc, based on the extent of skin fibrosis: (1) limited cutaneous SSc and (2) diffuse cutaneous systemic sclerosis. The latter is characterized by more extensive internal organ involvement. In addition, overlap syndromes of SSc with other connective tissue diseases and SSc without scleroderma (SSc without skin fibrosis) have been described [4].

The etiology of SSc is still unclear, but the clinical expression and the progression of the disease are due to genetic and environmental factors and to immune mechanisms. The pathogenesis of SSc is based on three features: vascular injury, immune system activation and generalized interstitial and vascular fibrosis [5]. The clinical heterogeneity of SSc is probably a reflection of the variable contribution of each pathogenic feature in different patients.

Cardiac disease in patients with SSc is due either to direct tissue damage (myocarditis, pericarditis, cardiac fibrosis, coronary artery disease, heart failure, arrhythmias) or indirect effects secondary to pulmonary hypertension or to renal disease. Cardiac manifestations may appear in all disease subsets, including in overlap syndromes, but tend to have a higher prevalence in the diffuse form [6,7].

The diagnosis of heart disease in SSc is often delayed because of a high variability of signs and symptoms, frequently attributed to non-cardiac pathologies (such as pulmonary, musculo-skeletal or esophageal disease). In addition, routine cardiac investigations such as electrocardiograms or standard echocardiography have low sensitivity in detecting early cardiac involvement. Cardiac computed tomography, single-photon emission computed tomography, positron emission tomography or cardiac magnetic resonance imaging (CMR) have higher sensitivity and specificity for detecting the subclinical structural and functional changes associated with SSc, but they are difficult to access and indicated only for complicated cases [8,9,10,11].

There are ongoing discussions and early-stage studies exploring the use of photon-counting computed tomography (PCCT) in cardiac imaging, including its application in detecting subclinical structural and functional changes in the myocardium. PCCT offers superior spatial resolution and improved image quality compared to conventional CT, making it particularly valuable for visualizing subtle cardiac abnormalities that are common in SSc, such as myocardial fibrosis and microvascular damage. PCCT could become a part of multimodal diagnostic approaches in systemic diseases such as SSc. PCCT is not yet widely available and studies using this technique are small and still attempting to validate the method. Studies with PCCT include the assessment of coronary artery disease and myocardial tissue characterization [12,13].

Tissue Doppler imaging (TDI), speckle tracking echocardiography (STE) and 4D cardiac echocardiography are non-invasive and more accessible and cost-efficient tools that allow a better detection of subclinical cardiac dysfunction in patients with SSc. Myocardial work, part of STE, evaluates LV performance, incorporating afterload determination using cuff blood pressure and consequently providing a more load-independent measure compared with global longitudinal strain [14].

There are many echocardiographic studies demonstrating the presence of subclinical diastolic dysfunction in patients with SSc generated by myocardial hypertrophy and fibrosis and by associated arterial hypertension, sleep apnea or renal dysfunction [15,16,17,18,19,20,21,22,23].

Clinical manifestations of left ventricular systolic dysfunction are not very frequent in SSc. Standard 2D echocardiography has detected systolic dysfunction with a left ventricular ejection fraction below 55% in 7% of SSc patients, but detection doubled after using tissue Doppler imaging [24].

Compared to other autoimmune diseases, such as rheumatoid arthritis and lupus erythematosus, systemic atherosclerosis seems to have a lower prevalence in SSc, with a less prominent inflammatory component and less aggressive manifestations, making the diagnosis of subclinical atherosclerosis more difficult [25,26]. In elucidating the prevalence of peripheral artery disease, the ankle brachial index (ABI) for the lower limbs, blood pressure difference between upper limbs, pulse wave analysis and velocity were used as investigation tools [27]. The cardiovascular ankle index (CAVI) is an easy to measure parameter of arterial stiffness, reflecting stiffness from the ascending aorta to the ankle arteries with little dependence on blood pressure [28]. In studies, it has been associated with the incident cardiovascular risk [29].

The current study started from the hypothesis that SSc causes microvascular damage and myocardial fibrosis and that over time, passing through a subclinical phase, permanent cardiac and vascular dysfunction occurs. Non-invasive complex cardiac ultrasound would detect cardiac dysfunction in its early, subclinical phase and identify cases at risk for overt disease. In addition, we wanted to quantify arterial stiffness by classical methods correlated with modern, feasible tools, such as the cardiovascular ankle index. Finally, but nevertheless important, we aimed to follow up on patients under SSc treatment in order to observe if standard therapies prevent the evolution of cardiac disease.

Our results validate the hypothesis by demonstrating that subclinical cardiac dysfunction in SSc can be detected early using advanced echocardiographic techniques and that standard therapies may prevent disease progression in the short to medium term. The findings pave the way for further longitudinal studies and the development of predictive risk scores to enhance the clinical management of SSc.

## 2. Materials and Methods

The study was conducted prospectively and included patients from the Rheumatology Department of the Clinical Hospital “Dr. Ioan Cantacuzino”, Bucharest, Romania. The study was conducted in accordance with the Declaration of Helsinki and approved by the local ethics committee (Clinical Hospital “Dr. Ioan Cantacuzino”). Informed consent was obtained from all subjects involved in the study.

**Figure 1:**
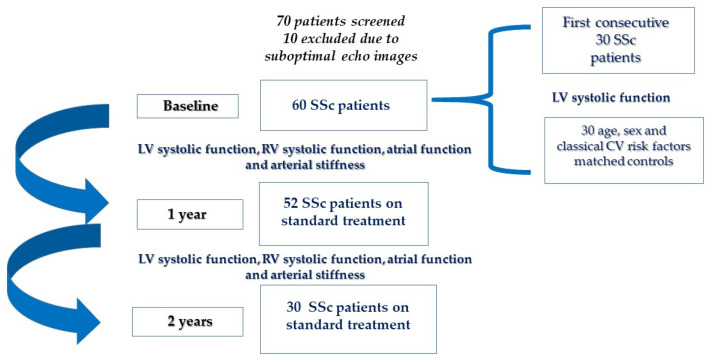
Study design.

A total of 70 consecutive patients with systemic sclerosis, presenting directly or through referral from other hospitals and fulfilling the ACR/EULAR 2013 classification criteria [30], were screened at baseline. Ten were excluded due to suboptimal ultrasound window. Sixty SSc patients were enrolled. The first 30 SSc patients enrolled were compared to a group of 30 age-, gender- and classical cardiovascular risk factor (smoking, diabetes, arterial hypertension, dyslipidemia, obesity)-matched and otherwise healthy controls (transversal study SSc patients vs. controls). Cardiovascular follow-up was conducted at 1 and 2 years (Figure 1) (longitudinal study: follow up of SSc patients).

Patients with other autoimmune connective tissue diseases, documented cardiovascular or cerebrovascular disease, chronic liver disease, chronic kidney disease, pulmonary disease, malignancy or pregnant women were excluded.

Baseline and follow-up visits included a clinical cardiovascular exam, ECG, cardiac ultrasound and evaluation of arterial stiffness. Also, the time since initial diagnosis and concomitant treatment was noted.

**Cardiac ultrasound** was performed using a GE VIVID E95 machine (GE Healthcare). Images were digitally archived for off-line analysis by a dedicated software (EchoPac BT012 version).

1. ***Conventional echocardiography*** was used for the assessment of the cardiac structure and function.
  1.1. For the **left ventricle systolic function**, we measured LVEF from the 4 and 2 chamber views using a biplane method of disks summation and lateral mitral annular plane systolic excursion measured by M-mode (MAPSE).
  1.2. For the **left atrium structure and function**, we measured the maximum and minimum volumes indexes using the area–length method.
  1.3. For the **right ventricle function**, we measured the RV systolic and diastolic areas by manual tracing of the endocardial border in order to calculate the fractional change area (FAC %), tricuspid annular plane systolic excursion measured by M-mode (TAPSE); pulmonary peak systolic pressure using tricuspid regurgitant jet velocity envelope (when feasible) and inferior vena cava (IVC) dimensions.
  1.4. For the **right atrium structure and function**: transversal diameter and volume index using area–length method in apical 4-chamber view.
2. ***On-line tissue Doppler echocardiography*** was used to measure the RV peak systolic velocity (S’) at the lateral tricuspid annulus level.
3. ***Two-dimensional speckle tracking echocardiography*** was used to calculate the percent of deformation (strain) for all cardiac chambers.
  3.1. After optimizing the frame rate, we manually traced the endocardial borders at the end-systole. *LV peak systolic global longitudinal strain (LV GLS)* was automatically calculated as the average of 18 segments from the 4-, 2- and 3-chamber views. We excluded from the analysis images with more than two inadequately visualized segments.
  3.2. *RV longitudinal strain (RV GLS)* was calculated as the average of the three segment deformations of the RV free wall in the apical 4-chamber view.
  3.3. *Left and right atrial deformation* were also assessed using a manual tracing of the endocardial border at the atrial end-systole in apical 4-chamber view. We calculated for both atria the peak negative strain (PNS), as marker of the active atrial function; peak positive strain (PPS), as marker of conduit function; and global strain (GS), as a marker of reservoir function.
  3.4. *The left ventricular myocardial work parameters* were automatically calculated during mechanical systole and isovolumetric relaxation (IVR): global constructive work (GCW), performed during shortening in systole adding negative work during lengthening in IVR; global wasted work (GWW), performed during lengthening in systole adding work performed during shortening in IVR; global work efficiency (GWE) as GCW/(GCW + GWW); and global work index (GWI), as the GCW + GWW.
4. ***Real-time 3D echocardiography*** was used to assess the *left ventricular ejection fraction and left ventricular strain* using the software 4DLVQ (“4D left ventricle quantification”) (EchoPAC PC version 108.1.4). The apical views were corrected to display standard 2-, 3-, and 4-chamber views. The end-diastolic frame was automatically detected from the ECG, and three points (two for the mitral plane and one for the left ventricular apex) were defined manually for each of the three views. The endocardial border was automatically traced and then manually adjusted for optimal tracing. Time–volume curves were generated.

**Arterial stiffness** was quantified using the **cardio-ankle vascular index (CAVI)** and **the ankle brachial index (ABI),** determined by an automatic vascular screening system (Fukuda Denshi VaSera VS-1500, Tokyo, Japan). We placed ECG electrodes on both wrists, a fonocardiogram microphone on the sternum and blood pressure cuffs on all limbs. Pulse wave velocity at the brachial and ankle levels, as well as blood pressure and ABI were automatically calculated concomitantly to CAVI. Results were reported for the left and right sides.

**Statistical analysis** was performed with SPSS software (version 20.0) (SPSS Inc., Chicago, IL, USA). Descriptive data are presented as mean value ± standard deviation. Unpaired Student’s *t*-tests and the analysis of variance (ANOVA) were used to compare the mean values. The independence of categorical variables was assessed using the chi-square test. The correlation coefficient for quantitative variables was assessed using Pearson’s correlation test and Spearman’s test. A value of *p* < 0.05 was considered statistically significant.

## 3. Results

### 3.1. Transversal Study: Left Ventricular Systolic Function in SSc Patients vs. Matched Controls

Sixty subjects (52 ± 9 years, 58 women) were studied: (a) 30 patients with SSc (55% limited, 45% diffuse cutaneous form), mean time since onset 6 ± 2 years, mean time since treatment start 2.5 ± 0.5 years, and (b) 30 age- and cardiovascular-risk-factor-matched otherwise healthy subjects (Figure 1).

Systemic sclerosis treatment was distributed as follows: 15% of patients were on corticosteroids, 25% were on Methotrexate or Azathioprine (for pulmonary, cutaneous, articular affectation), 20% were on Bosentan (for digital ulcers), 50% on calcium channel blockers (Amlodipine or Nifedipine, for peripheral vascular disease and/or high blood pressure) and 5% were on other vasodilators (Pentoxifylline).

The general characteristics of the two study groups are presented in Table 1. A total of 29 patients with SSc (out of 30) were females. Mean BMI was at the upper limit of normal. All patients were in NYHA class I, with normal blood pressure and heart rate. Regarding cardiovascular risk factors, none of the SSc subjects had diabetes, 10% were smokers and 20% had a total cholesterol level higher than 190 mg/dL. Cholesterol fractions were not available for all patients. As mentioned before, patients with SSc and overt cardio- or cerebrovascular disease, as well as severe pulmonary disease (including known PAH) were not included in the study. Patient co-morbidities were confirmed/infirmed at enrollment based on a complete medical history, medical exam, ECG and standard echocardiography.

The 2D standard echo parameters, including 2D ejection fraction, were similar between the two study groups (Table 2). Speckle tracking parameters and 4D echocardiography parameters, although in normal limits, were significantly altered in the SSc group. The parameters of myocardial work were similar in the two study groups, with the exception of global constructive work, which was significantly lower in SSc patients compared to controls (Table 3).

### 3.2. Longitudinal Study: Echocardiograhic and Vascular Follow-Up at One and Two Years

A total of 60 subjects (54 ± 11 years, 58 women) with SSc (74% limited, 26% diffuse cutaneous form, mean time since onset 6.2 ± 6.7 years, mean time since treatment start 2.0 ± 1.5 years) were studied at baseline. Of those, 52 subjects were reassessed after 1 year and 30 after 2 years of treatment (21% corticosteroids, 32% Methotrexate or Azathioprine, 26% Bosentan, 55% calcium channel blockers, 5% other vasodilators) (Figure 1). The general characteristics of the SSc patients at baseline are shown in Table 4. At follow-up at one and at two years, there were no significant differences from baseline regarding BMI, blood pressure, heart rate and NYHA class, as well as treatment plans.

A total of 58 patients with SSc (out of 60) were females. Mean BMI was at the upper limit of normal. All patients were in NYHA class I, with normal blood pressure and heart rate. None of the SSc subjects had diabetes, 15% were smokers and 24% had a total cholesterol higher than 190 mg/dL. The erythrocyte sedimentation rate was slightly above normal.

**Figure 2:**
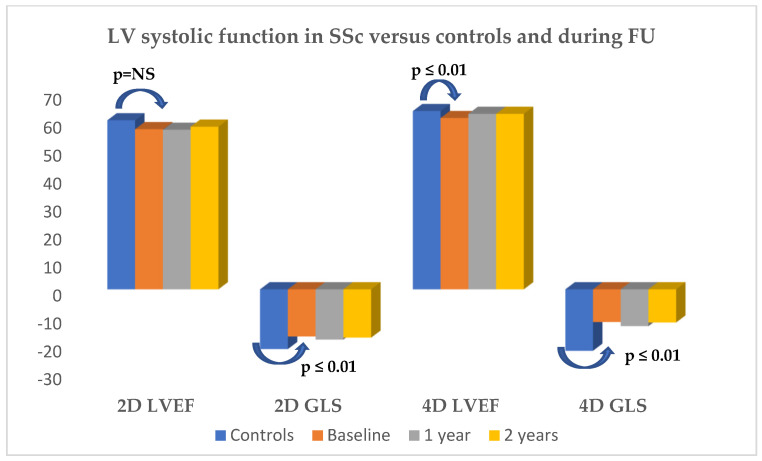
LV systolic function parameters in SSc patients vs. controls and during follow-up at one and two years. For follow-up, *p* = NS for all parameters.

At baseline, standard echo parameters were normal. As we have already shown, SSc patients have subclinical systolic dysfunction compared to age-, sex- and cardiovascular-risk-factor-matched controls (Figure 2). After 1 year and similarly after 2 years of treatment, 2D standard, TDI and speckle tracking echo parameters (for left and right ventricular function as well as atrial function), as well as 4D echocardiography and arterial stiffness parameters, were similar to baseline, with no correlation to the disease type and duration, treatment type, treatment duration or ESR value (Table 5, Figure 2).

## 4. Discussion

In this study, we assessed 2D and 4D cardiac ultrasound parameters of heart function in patients with different forms of SSc, without overt cardiovascular disease, versus controls. In addition, we monitored the evolution of cardiac function and arterial stiffness parameters under standard scleroderma therapy at one and two years. We had the following significant findings: 1. Patients with SSc have subtle subclinical left ventricular dysfunction, detectable by 2D speckle tracking parameters, 4D echography and myocardial work parameters, even if standard 2D echography parameters are normal. 2. Patients with treated SSc do not develop supplementary cardiac dysfunction (determined through standard and advanced echocardiography) or arterial stiffness in medium-term follow-up.

To our knowledge, this is one of the few studies that provides complex non-invasive cardiac and vascular function assessment and follow-up of SSc patients without known heart disease, comparing also the baseline characteristics to matched controls.

Overtly decreased left ventricular ejection fraction is a rare feature of SSc. In the EUSTAR database, Allanore et al. reported a prevalence of 5.4% of SSc patients with an LVEF under 55% [31]. A study including participants from the Australian Scleroderma Cohort study recorded 2.4% patients with an LVEF under 50% and only 0.6% with an LVEF under 40% [32]. The percentage of LVEFs under 45% in SSc is higher in studies using cardiac magnetic resonance [11].

Mitral annular plane systolic excursion (MAPSE) is an echocardiographic parameter used to assess longitudinal left ventricular function. It measures the displacement of the mitral annulus toward the apex during systole, reflecting the contraction of longitudinal myocardial fibers. In systemic sclerosis, MAPSE could be of particular interest due to its wide availability and sensitivity in detecting subclinical cardiac dysfunction before the alteration of the LV ejection fraction. In our study, MAPSE was similar in SSc patients and controls, and did not undergo significant alterations over time. Our literature research did not find a specific study evaluating MAPSE for the diagnosis of subclinical cardiac dysfunction; however, Berger et al. reported lower and subclinical values of MAPSE compared to controls for patients with mixed connective tissue disease (with clinical and biological features overlapping partly with other systemic diseases, such as systemic lupus erythematosus and systemic sclerosis) [33].

During the last two decades, new echocardiographic methods, such as tissue Doppler and speckle tracking echocardiography, were introduced for LV function evaluation in SSc in order to detect early dysfunction and to better stratify patients at risk for cardiovascular morbidity and mortality [34]. When LV global longitudinal strain is used, a higher number of asymptomatic SSc patients with normal LVEF values exhibit subclinical cardiac dysfunction. In 2018, a case–control, single-center study on 52 systemic sclerosis patients detected a significantly impaired left and right ventricular global longitudinal strain in patients with SSc when compared to controls (−19.2% vs. −21.1% for LV global strain and −18.2% vs. −22.3% for RV global strain), despite normal ejection fraction [35]. Tennøe et al. reported in 2019 a low global longitudinal strain (lower than −17.0%) in 47 of 192 SSc patients (24%) versus only 2 of 38 controls (5%) [36]. In 2024, the Australian Scleroderma Cohort sub-study detected an impaired LV global strain in 21% of patients, despite an LVEF ≥ 50% [32].

In our study, SSc patients had a normal LV ejection fraction determined through 2D echocardiography. However, when using more refined echocardiographic methods (such as 2D speckle tracking, myocardial work or 4D echocardiography), the parameters of LV function, although in normal limits, were decreased compared to matched controls.

Four-dimensional echocardiography and 4D cardiac strain are used more and more in clinical practice due to their high temporal resolution, minimal risk and relatively low cost. An accurate description of myocardial deformation by 4D echocardiography contributes to a better understanding of the mechanisms of cardiac dysfunction in SSc. In our study, 4D LVEF and 4D global longitudinal strain were significantly altered in SSc compared to controls.

Myocardial work evaluates LV performance by providing a more load-independent measure compared with LV global longitudinal strain [37]. In our research, global constructive work (GCW), the work performed by the ventricle that contributes to LV function during systole, was significantly lower in the SSc group versus controls. We know from other pathologies that a low GCW is associated with significant fibrosis on cardiac MRI [38]. Recent MRI studies have shown that myocardial fibrosis is also present in SSc patients. GCW could therefore become an accessible tool for detecting early myocardial fibrosis in SSc subjects [39].

Based on the data from the transversal study, our research is one of the few, if not the only one, which addresses the problem of SSc with normal LVEF but subclinical cardiac dysfunction, providing multiple parameters for quantifying subtle heart function changes and thus contributing to a more refined stratification of cardiac risk in these patients.

The evolution of heart function and of arterial stiffness parameters under standard rheumatological treatment in SSc has not been studied much. We wanted to observe if treated SSc patients develop subclinical or overt cardiac dysfunction on short- and medium-term follow-up. In our cohort, treatment was prescribed by rheumatologists based on the presence of joint lesions, skin disease, digital ulcerations or Raynaud syndrome. None of the patients were on active cardiovascular treatment. Calcium channel blockers were administered mainly for their vasodilation properties. At one year, as well as at two years of follow-up, we did not notice any significant modifications of LV systolic function, RV function or atrial function—determined through classical or new echocardiographic methods. Arterial stiffness parameters were within normal limits and stationary during follow-up.

Our data on LV systolic function are consistent with the results of similar small studies in the literature. Tennøe et al. did not find any deterioration of LV function measured through LVEF, LV GLS and the LV shortening fraction over a 3.3-year observation period [36]. The same authors linked this stability of LV function to the administration of calcium channel blockers (present in 55% of our study group), which may increase cardiovascular function. In a small study published in 2005, fourteen days of treatment with nifedipine in SSc patients improved myocardial perfusion and function, as evaluated by cardiac MRI and tissue Doppler echocardiography [40].

Our patients had normal values for RV systolic function (TAPSE, RV fractional area change, RV myocardial systolic velocities) and systolic pulmonary pressure at baseline and during follow-up. As RV function in SSc patients with or without pulmonary hypertension is much studied, it was not the focus of our research. Mean systolic pulmonary artery pressure was normal in our cohort and did not deteriorate over the 2 years of follow-up. We did not compare RV parameters to those of controls, unlike other studies [36,41,42,43] that report normal but lower values of RV parameters in systemic sclerosis. Nevertheless, our study is one of the few that provides follow-up of RV function in SSc without detecting significant deterioration over time. Tennøe et al. measured TAPSE as main parameter of RV systolic function at baseline and at 3.3 years and detected only a trend of TAPSE deterioration in SSc patients without pulmonary hypertension [36].

Studies on the left and right atrial function in SSc patients are few. Some authors have evaluated atrial function measured through speckle tracking echocardiography or cardiac MRI as a complementary parameter for diastolic function or as a mortality predictor [44,45]. We considered atrial function not only as a parameter linked to diastolic dysfunction but also a potential risk factor for atrial arrhythmias, which are frequent in SSc. Detection of atrial dysfunction could be therefore useful for mitigating arrhythmic risk. We assessed atrial structure (through indexed volumes) and function (through global strain measured by speckle tracking echocardiography), which were normal at baseline and during follow-up with a slight trend for LA global strain deterioration. Atrial function parameters did not correlate to mortality or supraventricular arrhythmic events, as our patients were all alive and event free at 2 years. LA global strain deterioration could be an indicator of fibrosis, but further follow-up and correlations with cardiac MRI are necessary in order to confirm this hypothesis.

Our study aimed to assess arterial stiffness and the presence of peripheral artery disease in SSc and to monitor parameters of arterial stiffness under standard treatment and during follow-up. As vascular markers we used the classic ABI but also the cardio-ankle vascular index (CAVI), which represents a novel parameter of arterial stiffness that does not depend on blood pressure. The CAVI values, according to age and gender, are classified as normal (CAVI < 8), borderline (8 ≤ CAVI < 9) and abnormal (CAVI ≥ 9) [46].

In our cohort, parameters of arterial stiffness were normal at baseline and did not alter during follow-up. We did not notice any correlation between vascular function and inflammation at baseline, but we had only ESR as marker of inflammation and ESR values were borderline for age in our cohort and could not be followed-up in all patients at 1 and 2 years in order to extend the correlation over time.

The presence of accelerated atherosclerosis in SSc remains unclear to date. Our data do not confirm the presence of macrovascular peripheral artery disease (defined by ABI) in patients with treated SSc, unlike other studies published in the last 3 decades. Youssef and colleagues demonstrated a five-fold increase in the prevalence of peripheral macrovascular disease, detected by peripheral angiography or Doppler ultrasonography, in 31 patients with SS compared to the control group [47]. The study by Ho et al. included 54 patients with SSc in the absence of other cardiovascular risk factors and detected a significantly increased prevalence of peripheral artery disease in SSc, defined as ABI < 0.9, compared to controls. In addition, two SSc patients developed acute ischemia of the limb that required amputation, with lesions similar to a classic atheromas at anatomopathological examination [48].

In addition, subclinical arterial stiffness (defined by CAVI) was absent in our cohort, consistent with other data from the literature. In 2017, Pussadhamma et al. investigated the correlations between CAVI and systolic pulmonary artery pressure at echocardiography in 145 SSc patients and found normal CAVI values and a correlation trend with the level of pulmonary arterial pressure in the diffuse SSc subgroup [49]. Also, another SSc study comparing arterial stiffness (defined through augmentation index and pulse wave velocity) in 14 SSc patients vs. controls demonstrated that SSc patients had greater augmentation index but lower PWV than the controls [50].

Our results on vascular function suggest that macro- and microvascular alterations occur later in the development of the disease and that they may be further delayed if the disease is controlled by standard therapy [51].

### Study Limitations

Our systemic sclerosis cohort is small, due to recruitment in a single rheumatology center and the rarity of the disease. Also, we could not compare the complete SSc cohort to a similar number of controls at baseline or during follow-up because of the difficulty in recruiting a high number of matched and otherwise healthy controls. Nevertheless, few studies on systemic sclerosis have a high number of patients and due to the exhaustive echocardiographic examination, our results are unique and a valuable add-on to the contemporary data on cardiac disease in SSc.

Patients came from different parts of the country, and some got lost during follow-up. However, we still have a significant number of patients who completed the visit at 2 years, making our study one of the few providing cardiac and vascular follow-up in the medium term in SSc patients without overt cardiovascular disease.

The study involved mainly women, but this reflects the general demographics of systemic sclerosis, which affects predominantly the female sex. The mean body mass index of the SSc patients was at the upper limit of normal, fitting the weight status of the population in our country.

A correlation with cardiac biomarkers, inflammation markers and specific antibodies at baseline and during follow-up would have been very useful for a better characterization of the cohort. This was not possible for all patients and the data are therefore not interpretable.

Our study group was heterogeneous regarding systemic sclerosis therapy due to the fact that patients were enrolled consecutively and received therapy as recommended by rheumatology guidelines. We think that it is a representative snapshot of the SSc population without documented atherosclerotic cardiac and cerebrovascular disease referred to a rheumatology department.

## 5. Conclusions

Patients with systemic sclerosis without overt cardiac and vascular disease have subtle cardiac dysfunction compared to age-, sex- and cardiovascular-risk-matched controls. Novel echocardiographic methods such as 2D speckle tracking echocardiography and 4D echocardiography are superior to standard echocardiography in detecting early changes. Myocardial global constructive work has special value and could become an echocardiographic marker for myocardial fibrosis in systemic sclerosis.

On medium-term follow-up, cardiac (systolic left and right ventricular and atrial) and macro- and microvascular functions remain in normal limits and do not deteriorate in patients on standard systemic sclerosis therapy, suggesting that subclinical changes appear early in the course of the disease and could be delayed by correct treatment. A longer follow-up and correlations with inflammation and specific antibody levels are needed in order to fully validate our hypothesis.

This is one of the few studies providing a complex and complete cardiac ultrasound profile in systemic sclerosis patients without documented cardiac involvement with follow-up of cardiac and arterial stiffness parameters at one and two years. More complex parameters should be included in the initial echocardiographic evaluation of systemic sclerosis patients. Based on these parameters, scores for detecting patients at high risk for cardiovascular morbidity and mortality will be developed.

## Figures and Tables

**Table 1:** General characteristics of SSc patients vs. matched controls.

Parameter	SSc (*n* = 30)	Controls (*n* = 30)
Age (yrs)	52.1 ± 9.5	51.6 ± 7.8
Female sex	1/30	1/30
BMI (kg/m^2^)	24.1 ± 3.7	26.7 ± 4.1
NYHA class	I	I
Systolic BP (mmHg)	123 ± 19	124 ± 16
Diastolic BP (mmHg)	75 ± 17	75 ± 12
HR (bpm)	68 ± 9	73 ± 11
Diabetes mellitus	0	0
Smoking (%)	10	10
Total cholesterol > 190 mg/dL (%)	20	20

SSc, Systemic Sclerosis; BMI, Body Mass Index; BP, Blood Pressure; HR, Heart Rate.

**Table 2:** Standard 2D echocardiography parameters in SSc patients vs. matched controls.

Parameter	SSc (*n* = 30)	Controls (*n* = 30)	*p* Value
Aortic annulus (mm)	23 ± 2.3	20.9 ± 2.1	NS
LA diameter (mm)	35.6 ± 6.5	34.1 ± 4.3	NS
IVS diastole (mm)	11.3 ± 1.4	11.2 ± 2.0	NS
LVPW diastole (mm)	11.2 ± 1.2	10.9 ± 1.8	NS
Indexed LVEDD (mm/m^2^)	22.9 ± 1.6	23.1 ± 2.1	NS
RA diameter (mm)	42.1 ± 7.6	44.4 ± 2.9	NS
RV diameter (mm)	22.7 ± 1.8	23.4 ± 2.0	NS
TAPSE (mm)	23.9 ± 4.2	24.2 ± 2.8	NS
SPAP (mmHg)	24.0 ± 7.5	22.9 ± 4.0	NS
2D LVEF (%)	57.4 ± 7.6	60.5 ± 5.1	NS
MAPSE (mm)	15.5 ± 3.3	15.7 ± 4	NS

SSc, systemic sclerosis; 2D LVEF, two-dimensional left ventricular ejection fraction; MAPSE, mitral annular plane systolic excursion; LA, left atrium; IVS, interventricular septum; LVPW, left ventricular posterior wall; LVEDD, left ventricular end diastolic diameter; RA, right atrium; RV, right ventricle; TAPSE, tricuspid annular plane systolic excursion, SPAP, systolic pulmonary artery pressure.

**Table 3:** STE and 4D echo parameters of systolic function in SSc patients vs. matched controls.

Parameter	SSc (*n* = 30)	Controls (*n* = 30)	*p* Value
2D GLS (%)	−17.1 ± 2.1	−21.4 ± 1.8	≤0.01
4D LVEF (%)	54.5 ± 8.5	63.8 ± 3.1	≤0.01
4D GLS (%)	−14.2 ± 2.4	−22.0 ± 2.7	≤0.01
GCW (mmHg %)	2124.2 ± 449.5	3102.8 ± 337.5	0.02
GWW (mmHg %)	81.0 ± 51.3	73.5 ± 38.9	NS
GWE (mmHg %)	95.0 ± 2.8	95.9 ± 2.1	NS
GWI (mmHg %)	1869.9 ± 410.9	2023.2 ± 321.7	NS

SSc, systemic sclerosis; 2D GLS, two-dimensional global longitudinal strain; 4D LVEF, four-dimensional left ventricular ejection fraction; 4D GLS, four-dimensional global longitudinal strain; GCW, global constructive work; GWW, global wasted work; GWE, global work efficiency; GWI, global work index.

**Table 4:** General characteristics of SSc patients at baseline.

Parameter	SSc
Age (years)	54.0 ± 11.0
Female sex	58/60
BMI (kg/m^2^)	24.1 ± 3.7
NYHA class	I
Systolic BP (mmHg)	125 ± 20
Diastolic BP (mmHg)	78 ± 16
HR (bpm)	69 ± 9
Diabetes mellitus	0
Smoking (%)	15
Total cholesterol > 190 mg/dL (%)	24
ESR (mm)	28 ± 18
Diffuse cutaneous form (%)	26
Limited form (%)	74
Times since onset (years)	6.2 ± 6.7
Time since treatment start (years)	2.0 ± 1.5
Corticosteroids	21%
Immunosuppressive therapy	32%
Bosentan	26%
Calcium channel blockers	55%
Pentoxifylline	5%

SSc, Systemic Sclerosis; BMI, Body Mass Index; BP, Blood Pressure; HR, Heart Rate; ESR, Erythrocyte Sedimentation Rate.

**Table 5:** LV, RV, atrial function parameters and arterial stiffness in SSc patients: follow-up.

Parameter	BL	1 Year	2 Year	*p* Value
**LV function**				
2D LVEF (%)	57.3 ± 7.0	57.1 ± 5.5	58.2 ± 4.4	NS
MAPSE (mm)	15.7 ± 3.3	15.4 ± 3.5	15.1 ± 3.2	NS
2D GLS (%)	−16.9 ± 2.7	−18.0 ± 2.2	−17.3 ± 3.2	NS
4D LVEF (%)	61.3 ± 6.5	62.8 ± 6.1	62.8 ± 6.1	NS
4D GLS (%)	−11.7 ± 4.2	−13.2 ± 3.4	−11.9 ± 4.3	NS
4D GAS (%)	−21.2 ± 6.3	−19.4 ±12.7	−21.3 ± 7.4	NS
**RV function**				
TAPSE (mm)	22.9 ± 3.4	22.4 ± 4.1	23.0 ± 3.6	NS
RV FAC (%)	35.1 ± 1.2	40.6 ± 1.0	35.8 ± 0.9	NS
RV GLS (%)	−21.0 ± 5.1	−19.6 ± 5.0	−19.2 ± 2.0	NS
SPAP (mmHg)	26.0 ± 10.3	29.4 ± 10.9	26.0 ± 7.5	NS
**Atrial function**				
LAVi (ml/m^2^)	22.3 ± 5.3	20. 4 ± 6.2	22.2 ± 4.5	NS
RAVi (ml/m^2^)	18.6 ± 7.0	19.0 ± 5.7	18.2 ± 6.0	NS
LAGS (%)	34.3 ± 4.4	26.2 ± 6.7	23.9 ± 8.0	NS
RAGS (%)	31.04 ± 9.3	29.5 ± 8.3	31.7 ± 7.8	NS
**Vascular function**				
CAVI	7.8 ± 1.0	7.6 ± 1.6	7.5 ± 1.3	NS
ABI	1.0 ± 0.1	1.0 ± 0.2	1.0 ± 0.1	NS

SSc, systemic sclerosis; 2D LVEF, two-dimensional left ventricular ejection fraction; MAPSE, mitral annular plane systolic excursion; 2D GLS, two-dimensional global longitudinal strain; 4D LVEF, four-dimensional left ventricular ejection fraction; 4D GLS, four-dimensional global longitudinal strain; 4D GAS, four-dimensional global area strain; TAPSE, tricuspid annular plane systolic excursion; RV FAC, right ventricular fractional area change; RV GLS, right ventricular global longitudinal strain; SPAP, systolic pulmonary artery pressure; LAVi, left atrial volume indexed; RAVi, right atrial volume indexed; LAGS, left atrial global strain; RAGS, right atrial global strain; CAVI, cardio-ankle vascular index; ABI, ankle brachial index.

## Data Availability

The raw data supporting the conclusions of this article will be made available by the authors upon request.
